# Lecanemab in DIAD: 6‐Month Amyloid PET Results from the DIAN‐TU‐001 Trial

**DOI:** 10.1002/alz70859_105303

**Published:** 2025-12-25

**Authors:** Kristin R Wildsmith, Arnaud Charil, Tammie L.S. Benzinger, Brian A. Gordon, Pratik Bhagunde, David Li, Guoqiao Wang, Larisa Reyderman, Jin Zhou, Eric McDade, Randall J. Bateman

**Affiliations:** ^1^ Eisai Inc., Nutley, NJ USA; ^2^ Washington University School of Medicine in St. Louis, St. Louis, MO USA; ^3^ Washington University School of Medicine, St. Louis, MO USA

## Abstract

**Background:**

The Dominantly Inherited Alzheimer Network Trials Unit (DIAN‐TU) adaptive platform trial has launched the first of three tau drugs in the tau Next Generation Prevention Trial (tau NexGen), starting with the combination of anti‐tau monoclonal antibody etalanetug (E2814) and anti‐amyloid‐beta antibody lecanemab. Herein, we provide the first results of lecanemab (open‐label) on amyloid PET in a symptomatic DIAD population (the asymptomatic cohort will be evaluated later).

**Methods:**

The DIAN‐TU‐001 Tau NexGen Trial (NCT05269394) is a phase II/III randomized, double blinded, placebo‐controlled trial in participants with DIAD. Eligible mutation‐positive participants were enrolled in 2 cohorts (Cohort 1: Symptomatic Population (CDR=0.5‐1), n=97; Cohort 2: Asymptomatic Population (CDR=0), n=100). The symptomatic Cohort 1 received open‐label lecanemab at Week 0 for 6 months and were then randomized to receive concurrent E2814 or placebo; meanwhile, asymptomatic Cohort 2 was randomized to E2814 or placebo at Week 0 and then received open‐label lecanemab at Week 52. For Cohort 1, amyloid PET ([^11^C]PiB‐PET) was performed at baseline (Week 0) and Week 24, and then at the weeks 52, 104, and 208 visits. Change on amyloid PET from Week 0 to Week 24 is a secondary endpoint for Cohort 1.

**Results:**

Preliminary Week 24 amyloid PET data was available from 62 symptomatic participants receiving lecanemab (avg EYO=1.51 and avg age= 47.4). Baseline mean amyloid PET SUVr was 2.06 (SD=0.38) (equivalent to 99.9 (SD=39.5) Centiloids (CL)) and SUVr=3.63 (SD=0.99) with partial volume correction (PVC). The change from baseline in amyloid PET SUVr at 24 weeks was ‐0.18 (SD=0.17) (equivalent to ‐18.6 CL (SD=17.3)) and SUVR=‐0.43 (SD=0.49) with PVC. The six‐month amyloid clearance results in participants with DIAD are consistent with pharmacokinetic (PK)/pharmacodynamic (PD) modeling in sporadic Alzheimer’s disease, with faster rate of amyloid clearance in older patients and those with lower baseline amyloid.

**Conclusions:**

Lecanemab reduced amyloid PET in symptomatic participants with DIAD at Week 24. The reduction in amyloid PET was in line with predictions based on PK/PD modeling developed from sporadic AD.

